# Predicting Coronary Artery Lesion Severity using Pulse Wave Harmonics: A SYNTAX Score-based Study

**DOI:** 10.2174/011573403X400522250519073551

**Published:** 2025-05-19

**Authors:** Haitian Li, Buxing Chen, GinChung Wang, Yunxiao Wang, Yang Yang

**Affiliations:** 1 Beijing University of Chinese Medicine, Beijing, 100029, China;; 2 Beijing University of Chinese Medicine Third Affiliated Hospital, Beijing, 100029, China;; 3 JinMu Health Technology, Taipei, Taiwan

**Keywords:** Arterial pressure wave, harmonics, SYNTAX score, degree of coronary artery lesions, photoplethysmography, ROC curve

## Abstract

**Introduction:**

This study aimed to investigate the correlation between the differences in pulse wave harmonic indices between the left and right hands and the SYNTAX score and to explore the potential of pulse wave harmonics in predicting the degree of coronary artery lesions.

**Methods:**

The arterial pressure wave signals from both hands of the patients scheduled for coronary angiography were recorded using photoplethysmography. According to the "visceral resonance theory", taking integer multiples of the heartbeat from 0 to 11 as the resonance frequencies, the collected arterial pressure waves were decomposed into the 0^th^ to 11th harmonics *via* the Fourier transform method. The harmonic characteristics were quantified by amplitude (Cn), phase (Pn), and energy (Dn) (n is the harmonic serial number), and the coefficient of variation of the indices was calculated and suffixed as CV. The difference between the measured values of the left- and right-hand parameters of the same patient was calculated (ΔCn,ΔPn,ΔDn,ΔCnCV,ΔPnCV), and the absolute value of the difference was obtained (|ΔCn|, |ΔPn|, |ΔDn|, |ΔCnCV|, |ΔPnCV|). Based on the coronary angiography imaging data, SYNTAX scores were computed for all participants, who were stratified by gender into male and female cohorts. For each group, logistic regression models were established with SYNTAX score≥22 as the dependent variable, and harmonic index differences as the independent variables. To determine the best prediction model, the Akaike Information Criterion (AIC) was used and model with the lowest score was selected. Finally, the discriminant ability of the prediction model was evaluated using the ROC curve analysis and the Bootstrap internal validation method.

**Results:**

A total of 348 patients were included, with 249 males and 99 females. In the male group, the discriminant model was based on |ΔC10|,ΔD6, |ΔD9|, |ΔD10|, |ΔP8|, |ΔP10|, ΔP1CV, andΔC9CV, with the minimum AIC value of 105.47, the area under the ROC curve (AUC) of 0.89, and the average AUC of 0.85 in the Bootstrap internal validation. In the female group, the discriminant model was based on |ΔD2|, |ΔD3|, |ΔD5|, |ΔD6|, |ΔD9|, |ΔC2CV|, |ΔC4CV|, |ΔC5CV|, |ΔC6CV|, and |ΔC9CV|, with the minimum AIC value of 59.34. The AUC of the ROC curve of this prediction model was 0.92, and the average AUC in the Bootstrap internal validation was 0.84.

**Discussion:**

In this study, the degree of coronary artery occlusion was evaluated through a noninvasive method combined with the SYNTAX score, providing a valuable noninvasive tool for clinical evaluation of CAD. This detection method is easy to operate, has high repeatability, and the equipment is small in size, making it suitable for various environments, it can be operated independently by the patients. Yet, the current study, being cross-sectional, only found an association rather than a causal relationship, calling for future prospective studies to clarify the causal link.

**Conclusion:**

The different characteristics of pulse wave harmonics between the left and right hands can effectively reflect the degree of coronary artery lesions. Through the analysis of pulse wave harmonics, a diagnostic model with good discriminant ability for predicting the degree of coronary artery lesions can be constructed, which may offer a valuable non-invasive tool for the clinical assessment of CAD.

## 1 INTRODUCTION

Coronary Heart Disease (CHD), a leading cause of mortality worldwide, remains a critical threat to global health [1]. Accurate assessment of the degree of coronary artery lesions plays a crucial role in guiding the formulation of treatment strategies and predicting patient prognosis [2]. Current clinical evaluation primarily relies on invasive coronary angiography and Coronary Computed Tomography Angiography (CCTA). However, these methods require specialized equipment, involve technical complexity, and pose risks, such as radiation exposure and contrast agent allergies. These limitations hinder its widespread clinical application. Consequently, there is an urgent need for a non-invasive, rapid, and safe alternative to assess coronary artery lesions.

The human body, as a complex and sophisticated system, contains rich biological information, such as electrocardiogram information, electroencephalogram information, and arterial pressure wave information. These pieces of information, like hidden codes, can reflect the physiological and pathological states of the human system or organs [3, 4]. Among these, arterial pressure wave information can be obtained through Photoplethysmography (PPG). Compared with other detection methods, its detection equipment is small in size and easy to operate, and it is more convenient to collect in clinical practice, which provides great convenience for clinical application. Building on the "visceral resonance theory" proposed by Professor Wang Weigong, which posits that blood flow between the heart and arterial system operates through harmonic resonance, PPG-derived pulse waves can be decomposed into harmonic components (0^th^-11^th^ order) using Fourier transform. These harmonics are quantified by amplitude (Cn), phase (Pn), and energy (Dn), forming the basis of arterial pressure wave harmonic analysis—a method proven to reflect systemic pathophysiological states, including cardiovascular function [5, 6]. Notably, prior studies have identified significant inter-hand differences in these harmonic indices among CHD patients. This study intends to deeply analyze the correlation between left-right hand arterial pressure wave harmonics and the coronary SYNTAX scores, aiming to explore the feasibility of the arterial pressure wave harmonic analysis method in evaluating the degree of coronary artery lesions. It is expected to open up a new and efficient path for the clinical evaluation of the degree of coronary artery lesions, addressing the limitations of current methods while offering high clinical significance and application value.

## 2 MATERIALS AND METHODS

### 2.1 Research Subjects

The patient data was collected from the Department of Cardiology, the Third Affiliated Hospital of Beijing University of Chinese Medicine, from February 2023 to May 2023. It was a single-center, cross-sectional observational study approved by the Ethics Committee of the Third Affiliated Hospital of Beijing University of Traditional Chinese Medicine, with the approval number BZYSY-2022KYKTPJ-09. All the studies were performed with the patients’ informed consent. All procedures involving human subjects were conducted in accordance with the Declaration of Helsinki. This study follows the STROBE and SAGER (Sex and Gender Equity in Research) guidelines for reporting. The choice of sample size is based on a scientific relationship based on a 1:10 ratio between logistic regression parameters and sample size. All data are securely stored in the hospital's electronic system. Under compliance with ethical approval and data protection regulations, the corresponding author can provide the anonymized data upon reasonable request.

### 2.2 Inclusion Criteria

Included patients had to meet the following conditions simultaneously:

1. Patients who have been diagnosed or are suspected to be diagnosed with coronary heart disease.
2. Patients scheduled to undergo coronary angiography.
3. Patients aged 18 years or older.
4. Patients who voluntarily participate in the study.

### 2.3 Exclusion Criteria

Patients meeting any of the following conditions were excluded:

1. Patients with concomitant severe diseases of the respiratory, urinary, digestive, hematopoietic, endocrine, and metabolic systems, as well as other mental and neurological disorders.
2. Pregnant or lactating patients.
3. Patients for whom pulse diagnosis measurements are not feasible.
4. Patients with peripheral vascular diseases or arteriosclerosis of the upper extremities.

### 2.4 Arterial Pressure Wave Measurement

Before all patients undergo coronary angiography, arterial pressure wave data were collected using a high-precision intelligent pulse acquisition device (Model HMYH1300, Jinmu Health Technology) to ensure an accurate reflection of the patients’ baseline physiological status at that time. Participants were positioned supine with hands resting naturally at their sides in a quiet environment to minimize external interference. Measurements were taken simultaneously from the index fingers of both hands to guarantee synchronized and consistent data acquisition.

### 2.5 Coronary Angiography and SYNTAX Score Calculation

Coronary angiography was performed using a Siemens large C-arm angiography machine (Model Artis zee floor), and iodixanol injection was selected as the contrast agent. Two independent cardiologists evaluated angiographic images to calculate SYNTAX scores according to the established criteria [7]. If the difference between the scores given by the two doctors was less than 5 points, the SYNTAX score of the patient was taken as the average of the two scores. If the difference between the scores was greater than 5 points, a cardiovascular specialist with a higher professional title (the third doctor) was involved to re-score, ensuring the accuracy and reliability of the SYNTAX score.

### 2.6 Calculation Method of the Difference Index of Arterial Pressure Wave Harmonics between the Left and Right Hands

The collected arterial pressure wave data were subjected to Fourier transformation. Taking integer multiples of the heartbeat as the resonance frequencies, the arterial pressure waves were finely decomposed into the 0^th^ to 11^th^ harmonics. For each harmonic, its amplitude (Cn), phase (Pn), and energy (Dn) were calculated, respectively. These parameters can quantitatively describe the characteristics of the harmonics. Meanwhile, to further analyze the degree of data dispersion, the coefficient of variation of each index was calculated, and "CV" was uniformly added after the corresponding index name to indicate it. Difference between the measured values of the left- and right-hand parameters of the same patient (ΔCn,ΔPn,ΔDn,ΔCnCV,ΔPnCV) were calculated, and the absolute value of the difference (|ΔCn|, |ΔPn|, |ΔDn|, |ΔCnCV|, |ΔPnCV|) was obtained. The calculation formulas for the harmonic indices are shown in Fig. (**1**).

### 2.7 Statistical Methods

The Matlab 2024b software was employed to perform comprehensive and systematic data processing. Participants were stratified by gender (male: n = 249; female: n = 99) to account for sex-specific physiological variations. Multivariable logistic regression models were developed for each group, with SYNTAX score ≥22 as the dependent variable, and inter-hand harmonic index differences (amplitude [Cn], phase [Pn], energy [Dn], and their coefficients of variation [CnCV, PnCV, DnCV]) as the independent variables. A parameter-to-sample ratio of 1:10 ensured statistical adequacy for the cohort size (N = 348). On this basis, a Receiver Operating Characteristic (ROC) curve analysis was further carried out to deeply explore the performance of the models. To construct the most effective prediction model, three classic modeling methods, namely the Forward selection method, the Backward elimination method, and the Stepwise regression method, were adopted during the research process. By comparing the Akaike Information Criterion (AIC) values of the models constructed using different methods, the model with the smallest AIC value was selected as the optimal model. As a widely used criterion for model selection, the AIC can find the best balance between the goodness-of-fit of the model and its complexity, ensuring that the selected model can accurately fit the data and has good generalization ability. The constructed prediction model was comprehensively evaluated from two key dimensions: discrimination and calibration. In terms of discrimination evaluation, the Area Under the Curve (AUC) of the Receiver Operating Characteristic (ROC) curve was used as the evaluation index. The AUC value can intuitively reflect the ability of the model to distinguish between the different categories of samples. In this study, an AUC statistic > 0.7 was used as the standard to determine that the model had good discrimination ability, thereby measuring its effectiveness in distinguishing between patients with a SYNTAX score≥22 and those with a SYNTAX score <22. For the calibration evaluation, the Bootstrap method was used to perform 2000 resamplings, and a calibration curve was drawn through internal validation to accurately evaluate the consistency between the predicted and the actual results. Meanwhile, the Hosmer-Lemeshow goodness-of-fit test was used to evaluate the calibration quantitatively. This test can determine the degree of fit between the predicted probabilities of the model and the actual observed results from a statistical perspective, ensuring that the model has high accuracy and reliability in the prediction process.

## 3 RESULTS

### 3.1 General Information

A total of 348 patients were included in this study, among whom 249 were male (average age 59.80), and 99 were female (average age 67.52). In the male patient group, 217 patients had a SYNTAX score of less than 22, and 32 patients had a SYNTAX score of 22 or higher. Among the female patients, 86 patients had a SYNTAX score of less than 22, and 13 patients had a SYNTAX score of 22 or higher.

### 3.2 Results of the Multivariate Regression Analysis Model and the ROC Curve Analysis for the Difference Values of Arterial Pressure Wave Harmonic Indices between the Left and Right Hands in the Male Group

The analysis results demonstrated that the overall model was statistically significant (*P* = 0.00), and among various attempts at model construction, the AIC value of this model was the smallest, at 105.47. This indicates that this model has achieved the best balance between goodness-of-fit and complexity, and has good reliability and generalization ability. Further analysis revealed that the correlations between the |ΔC10|, ΔD6, |ΔD9|, |ΔP8|, |ΔP10|, ΔP1CV, ΔC9CV and a SYNTAX score≥22, were statistically significant the detailed data are shown in Table **1**. In terms of model performance evaluation, through the ROC curve analysis, the AUC of this model was obtained as 0.88, refer to Fig. (**2**) for details. This result intuitively demonstrates that the model has a strong ability to distinguish between patients with different SYNTAX score levels. The Hosmer-Lemeshow goodness-of-fit test was used to evaluate the calibration of the model, and the calibration value obtained was 4.32 (*P* = 0.82), indicating a high degree of fit between the predicted probabilities of the model and the actual observed results. In addition, through the Bootstrap internal validation method, after multiple repeated sampling verifications, the results proved that the AUC was 0.85, with a confidence interval of [0.80, 0.87], further confirming the stability and reliability of the model results.

### 3.3 Results of the Multivariate Regression Analysis Model and the ROC Curve Analysis for the Difference Values of Arterial Pressure Wave Harmonic Indices between the Left and Right Hands in the Female Group

Logistic regression analysis was rigorously conducted with the difference values of the arterial pressure wave harmonic indices between the left and right hands of female patients, namely |ΔD2|, |ΔD3|, |ΔD5|, |ΔD6|, |ΔD9|, |ΔC2CV|, |ΔC4CV|, |ΔC5CV|, |ΔC6CV|, and |ΔC9CV|, as the independent variables, and a SYNTAX score ≥ 22 as the dependent variable. The analysis suggested that the overall model was statistically highly significant (*P* = 0.00). Among the comparisons of numerous model construction schemes, the AIC value of this model was the smallest, at 59.34. This result clearly indicates that when fitting the data, this model can accurately conform to the characteristics of the samples and has excellent generalization performance, achieving an ideal balance between the goodness-of-fit and the complexity of the model. Upon in-depth analysis of the model, it was further determined that there were statistically significant associations between the indices of the difference values, such as |ΔD2|, |ΔD5|, |ΔD6|, |ΔD9|, |ΔC5CV|, |ΔC6CV|, and |ΔC9CV| and a SYNTAX score ≥ 22 (Table **2**). During the comprehensive evaluation of the performance of this model, using the ROC curve analysis method, the AUC of this model was as high as 0.92, see Fig. (**3**) for details. This value intuitively and powerfully demonstrates that this model has excellent discrimination ability for female patients with different SYNTAX score levels and can accurately identify the group of patients with a SYNTAX score ≥ 22. In order to further evaluate the consistency between the predicted probabilities of the model and the actual observed results, the Hosmer-Lemeshow goodness-of-fit test method was used to evaluate the calibration of the model quantitatively, and finally, the calibration value obtained was 3.03 (*P* = 0.93). This result indicates that there is a very high degree of fit between the predicted values of the model and the actual observed values, as well as the predicted results of the model are highly reliable. In addition, by means of the Bootstrap internal validation method, after 2000 repeated sampling verifications, the final average area under the ROC curve was 0.84, with a confidence interval of [0.72, 0.91]. This result further validates the stability and reliability of the model results. Even under different sampling situations, the model can still maintain relatively stable and accurate prediction performance.

## 4 DISCUSSION

The SYNTAX score, proposed by the American College of Cardiology/American Heart Association (ACC/AHA), is a comprehensive and accurate tool for assessing the degree of coronary artery stenosis and guiding the selection of coronary revascularization methods, and it has been widely applied in clinical practice [8]. This scoring system constructs a risk stratification scoring system based on the anatomical characteristics of coronary artery lesions. Compared with other scoring criteria, it can more comprehensively demonstrate the anatomical features of coronary artery lesions, such as location, severity, bifurcation, and calcification. In view of this, the SYNTAX score was selected as an objective indicator for quantitatively evaluating the degree of coronary artery lesions in patients included in this study [9]. The SYNTAX score threshold of ≥22 is not only a widely accepted marker of complex coronary artery disease but also a critical predictor of adverse clinical outcomes. In the original SYNTAX trial, patients with SYNTAX scores≥22 demonstrated significantly higher rates of Major Adverse Cardiac Events (MACE) after Percutaneous Coronary Intervention (PCI), compared to Coronary Artery Bypass Grafting (CABG), establishing this threshold as a key determinant to guide revascularization strategies [7, 8]. Subsequent studies further validated its prognostic relevance; for example, SYNTAX scores≥22 correlate with increased risks of mortality, myocardial infarction, and repeat revascularization [2, 10]. The 2018 ESC/EACTS Guidelines on Myocardial Revascularization explicitly recommend using SYNTAX score≥22 to identify patients with complex lesions who may benefit more from CABG than PCI [11]. Thus, this threshold serves as both an anatomical severity indicator and a prognostic tool, aligning with its application in our study to stratify high-risk populations.

Studies have indicated that some cardiovascular diseases can cause asymmetry in the PPG waveform, especially between the finger signals of the left and right hands. This asymmetry is associated with various cardiac abnormalities, including Coronary Atherosclerotic Heart Disease (CAD). Although the mechanism underlying this asymmetry has not been fully elucidated, factors such as impaired circulatory function, vascular dysfunction, or autonomic nervous imbalance are speculated to be the possible causes [12].

Fourier transform, a commonly used method in physics for analyzing complex waves, is based on the core theory that a complex wave is composed of multiple simple waves. These simple waves can be separated from the complex wave through specific frequencies, and then the characteristics of the simple waves can be studied to analyze the complex wave [13]. As early as 1991, Wang *et al.* [5] proposed the visceral resonance theory, which states that the hemodynamic interactions between the heart and arterial system resonate at integer multiples of the fundamental heart rate frequency. The arterial pressure wave can be decomposed into multiple simple waves through Fourier transform. These component waves contain state information about various physiological systems within the human body, which can be used to study organ diseases or assess functional status. Since the 0^th^ to 11^th^ harmonics have been shown to contain over 98% of the energy of the arterial pressure wave [14], these twelve harmonics can effectively characterize the features of human blood circulation. From a pathophysiological perspective, the severity of coronary artery stenosis is closely related to myocardial ischemia. Changes in myocardial ischemia severity can induce corresponding abnormal cardiac motion alterations, which subsequently lead to variations in arterial pressure waveforms. Subsequently, statistical criteria were applied to refine the model. All 12 harmonics (C0–C11, P0–P11, D0–D11), and their CV were initially included as candidate variables. To avoid overfitting, a stepwise regression approach guided by the AIC was employed to select the most parsimonious set of predictors. Variables with significant contributions (*P*<0.05) to the prediction of SYNTAX score≥22 were retained in the final model. This dual approach—combining physiological theory and statistical rigor—ensured that the selected harmonic indices were both biologically plausible and empirically robust.

A total of 348 patients were included in this study. Based on the relationship between the logistic regression parameters and the sample size at a ratio of 1:10, the number of parameters used in the logistic regression equations, established in this study to determine whether the SYNTAX score was greater than or equal to 22 points, was less than 10 times the sample size. Therefore, the sample size was statistically sufficient. The study was divided into a male group and a female group mainly because previous studies have found significant differences between males and females in multiple harmonic indices.

As a non-invasive detection method, photoplethysmography can reflect changes in pulse pressure. Iqbal *et al.* [15] used the PPG technique to record six body sites of 51 controls and 20 patients with Systemic Sclerosis (SSc). The results showed that the classification accuracies under the two algorithms of EfficientNetB0 and GoogLeNet reached 87.3% (77.2-94.0) and 83.1% (72.3-90.9), respectively. Weng *et al.* [16] also processed the PPG signals of 214 patients with Peripheral Arterial Disease (PAD). The results indicated that the overall test sensitivity was 86.6%, the specificity was 90.2%, the accuracy was 88.9%, and the Cohen's Kappa statistic was 0.76. The sensitivity for mild-to-moderate disease was 83.0%, and the sensitivity for major disease was 100.0%. Numerous studies have demonstrated the strong potential of PPG in disease detection. The detection instrument is similar to a pulse oximeter, and has the characteristics of being portable and requiring a short detection time (only 1 minute) [17]. The data collected through the internet can be directly uploaded to the computing system, and the results can be fed back to the doctor's terminal, such as a mobile phone, enabling detection at any time, which is convenient, fast, and safe. In addition, this detection method is easy to operate, has high repeatability, and the equipment is small in size, making it suitable for various environments, and it can even be operated by patients themselves. In contrast, other methods for detecting pulse waves face problems, such as difficulty in operation and promotion, and accuracy that is easily affected by various factors. Therefore, this study has exploratorily revealed the potential application value of the analysis method for the difference values of arterial pressure wave harmonic indices in evaluating the degree of coronary artery lesions. In the future, by further increasing the research sample size and comparing with the results of the radial artery intravascular pressure wave detection method, a data diagnostic model is expected to be constructed to evaluate the degree of coronary artery lesions based on the arterial pressure wave indices, using the harmonic analysis method, opening up a new path for the diagnosis and treatment of coronary heart disease, and contributing to the improvement of the diagnosis and treatment level of cardiovascular diseases.

Beyond pulse wave harmonics, recent studies highlight the value of integrating myocardial and arterial functional markers, such as Ventricular-Arterial Coupling (VAC), for evaluating cardiovascular health. VAC, defined as the ratio of arterial elastance (Ea) to left ventricular end-systolic elastance (Ees), reflects the efficiency of energy transfer between the heart and arterial system. Noninvasive assessment of VAC *via* transthoracic echocardiography has emerged as a feasible approach to quantify cardiovascular performance, particularly in patients with Stable Ischemic Heart Disease (SIHD). Elevated baseline VAC, driven by reduced Ees and increased Ea, has been associated with impaired myocardial efficiency and adverse outcomes in SIHD cohorts [18]. Notably, Percutaneous Coronary Intervention (PCI) has been shown to improve VAC through enhanced Ees, suggesting its role in monitoring therapeutic efficacy [19]. The integration of pulse wave harmonic analysis with VAC assessment may offer a multimodal framework for risk stratification. While harmonic differences capture vascular impedance and asymmetry in peripheral circulation, VAC provides a systemic view of cardiac-arterial interactions. This combined approach could improve the detection of subclinical myocardial dysfunction, and refine prognostication in coronary artery disease. Future studies should explore the synergistic potential of these noninvasive markers, particularly in populations where invasive angiography remains impractical.

In this study, observations and measurements were conducted on the research subjects at a specific time point. An association was found between pulse wave harmonics and the degree of coronary artery lesions. Moreover, the analysis based on the SYNTAX score demonstrated that harmonics have good discriminative ability in predicting the degree of coronary artery lesions, suggesting that pulse wave harmonics might serve as an indicator to predict the degree of coronary artery lesions. However, this association does not equate to a causal relationship. To clarify a causal relationship, future prospective studies are required. These studies should continuously follow up on the research subjects, and observe the changes in pulse wave harmonics and the degree of coronary artery lesions over time, thereby determining the causal link between the two.

This study has certain innovative and clinical application value, however, there are some limitations.

Sample Size and Generalizability: Although 348 patients were included, the sample size may still be relatively small, potentially limiting the generalizability of the results to the entire population. Additionally, the patients were all from a single hospital, which may introduce selection bias, and the findings may not be applicable to other regions or patient populations with different characteristics.

Lack of Longitudinal Data: This study was cross-sectional, lacking longitudinal data to observe the dynamic changes of arterial pressure wave harmonics and SYNTAX scores over time. Thus, it is unable to establish a causal relationship between the two and cannot predict the long-term progression of coronary artery lesions. Potential Confounding Factors: Despite excluding patients with certain comorbidities, there may still be other unaccounted confounding factors, such as lifestyle factors (diet and exercise), genetic factors, and environmental factors, which could affect the arterial pressure wave harmonics and SYNTAX scores, and influence the accuracy of the model.

## CONCLUSION

This study demonstrates that left-right pulse wave harmonic differences correlate with SYNTAX score ≥22, supporting their potential as a noninvasive marker for coronary artery lesion severity. The gender-specific model had an AUC of 0.88 (male) and 0.92 (female), and the average AUC verified internally *via* Bootstrap was 0.85 and 0.84, indicating a strong discriminative ability. These findings provide a foundation for developing harmonic-based diagnostic tools to complement invasive angiography, particularly in resource-limited settings.

## Figures and Tables

**Fig. (1):**
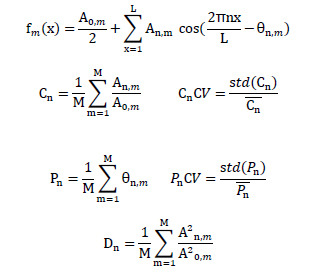
Calculation formulas for Cn, Pn, Dn, CnCV, and PnCV. **Note:** A(n, m) and θ(n, m) are the amplitude and phase of the n-th Fourier series of the m-th arterial pressure wave measured under a certain pressure. A(0, m) is the average value of the m-th arterial pressure wave. fm(x) is the x-th data point in the m-th arterial pressure wave. L is the total number of data points in Pm(x).

**Fig. (2):**
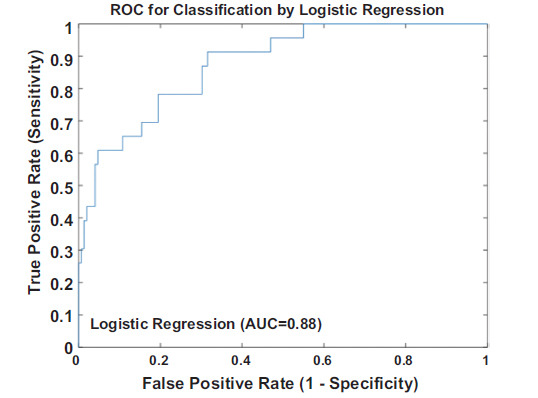
Results of the ROC curve analysis of the logistic regression model in the male group.

**Fig. (3):**
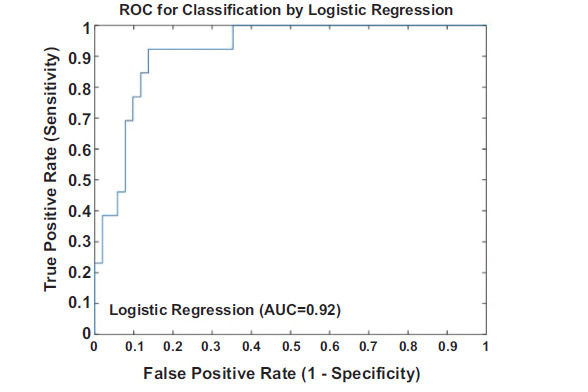
Results of the ROC curve analysis of the logistic regression model in the female group.

**Table 1:** Results of the logistic regression analysis between the harmonic difference indices of arterial pressure waves of the left and right hands and SYNTAX score ≥ 22 in male patients.

-	**Coefficient**	**SE**	**tStat**	***P* Value**
|ΔC10|	128.92	58.08	2.22	0.03*
ΔD6	4.94	1.96	2.52	0.01*
|ΔD9|	-15.90	5.41	-2.94	0.00*
|ΔD10|	5.69	3.07	1.85	0.06
|ΔP8|	4.10	1.43	2.86	0.00*
|ΔP10|	-2.73	1.04	-2.62	0.01*
ΔP1CV	61.86	24.43	2.53	0.01*
ΔC9CV	3.91	1.36	2.88	0.00*

**Note:** Coefficient refers to the fitting coefficient, SE represents the standard deviation, tStat is the statistical coefficient, and * indicates *P* < 0.05.

**Table 2:** Results of the logistic regression analysis between the difference values of arterial pressure wave harmonic indices of the left and right hands and SYNTAX score ≥ 22 in female patients.

**-**	**Coefficient**	**SE**	**tStat**	***P* Value**
|ΔD2|	-42.78	19.77	-2.16	0.03*
|ΔD3|	-32.48	16.66	-1.95	0.05
|ΔD5|	31.05	14.15	2.19	0.03*
|ΔD6|	-8.96	3.76	-2.38	0.02*
|ΔD9|	12.93	4.93	2.62	0.01*
|ΔC2CV|	-55.56	29.18	-1.90	0.06
|ΔC4CV|	-23.18	14.41	-1.61	0.11
|ΔC5CV|	16.95	8.06	2.10	0.04*
|ΔC6CV|	26.27	10.36	2.54	0.01*
|ΔC9CV|	-9.95	4.64	-2.14	0.03*

**Note: ** Coefficient refers to the fitting coefficient, SE represents the standard deviation, tStat is the statistical coefficient, and * indicates *P* < 0.05.

## Data Availability

The authors confirm that the data supporting the findings of this research are available within the article.

## LIST OF ABBREVIATIONS

AIC: Akaike Information Criterion
AUC: Area Under the Curve
CABG: Coronary Artery Bypass Grafting
CCTA: Coronary Computed Tomography Angiography
CHD: Coronary Heart Disease
MACE: Major Adverse Cardiac Events
PPG: Photoplethysmography
ROC: Receiver Operating Characteristic
VAC: Ventricular-arterial Coupling

## STANDARDS FOR REPORTING

STROBE and SAGER guidelines were followed.
